# Recording of patient weight in the intensive care unit: a survey of current practice

**DOI:** 10.1186/2197-425X-3-S1-A918

**Published:** 2015-10-01

**Authors:** S Birkholzer, N Richardson, E Fitzgerald, B Harris, J Knighton

**Affiliations:** Portsmouth Hospital NHS Trust, Department of Critical Care, Portsmouth, United Kingdom; University of Southampton NHS Foundation Trust, Department of Critical Care, Southampton, United Kingdom

## Introduction

A recent single-centre audit uncovered inadequacies in the practice of measuring and recording of patient body weight (BW), and knowledge of weight based clinical calculations in ICU. To further investigate this, we distributed and promoted a multi-disciplinary survey to assess current practice and knowledge in all ICU's in the Wessex region, UK.

## Objectives

The aim was to identify areas of good practice and implement this practice in all ICUs in the Wessex region.

## Methods

A survey was distributed to the multidisciplinary team in 12 ICUs. The following were included: responder job position, practice for weight and height recording in their ICU, and knowledge of what type of BW measurement should be used for a variety of drug dosing and lung protective ventilation (LPV) calculations.

## Results

451 responses were received. Nurses 60% (n = 270), Doctors in training 22% (n = 98), Consultants 13% (n = 57) and other healthcare professionals 3.5% (n = 16), 10 did not state their occupation. 48% of respondents replied that BW documented on admission was estimated. When BW was actually measured or calculated, 69% used actual BW (ABW) and 28% ideal BW (IBW), some both. The recorded height was measured by 52% of responders, estimated by 31% and derived from forearm or tibia length by 20%.

57% of responders correctly identified that IBW or predicted BW should be used for LPV. 21% of consultants answered incorrectly, 18% did not answer.

Exceptional good practice was demonstrated in one institution where BW was measured or calculated by 100% of 40 responders. In this ICU 100% of respondents knew that either ideal or predicted BW in used to calculate desired tidal volume as part of LPV.

When analysing answers to the drug dosing questions, only doctors and pharmacists were included (n = 161). 35% (n = 56) correctly indicated that ABW should be used to calculate Suxamethonium dosage in obese patients.53% (n = 86), 37% and 36% knew that dosing of unfractionated heparin, Gentamycin and Vancomycin respectively in non-obese patient is based on the ABW.

## Conclusions

Measurement and recording of accurate BW and height is inconsistent and poor in most of the ICUs surveyed. There are significant knowledge gaps in identifying what BW should be used for a variety of clinical and pharmacological drug calculations. Errors and lack of knowledge may result in ineffective treatment and unsafe quantities of therapeutic interventions like LPV.

Following this survey, compliance with LVP was audited in these ICUs. It demonstrated that in the institution where accurate documentation of BW and height is mandatory on admission, the compliance with LVP at 8 ml/kg was significantly better than any ICU in Wessex (100% compliance).

Local leads have presented the results of the survey and promoted documentation of ABW and height as a mandatory part of ICU admission. Following the education process, compliance with documentation of measured BW and height will be audited.

